# Screening of probiotics for promoting mineral absorption based on *in vitro* fermentation and cell models

**DOI:** 10.3389/fmicb.2026.1743657

**Published:** 2026-02-26

**Authors:** Bin Liu, Yuejian Mao, Jing Yang, Linjun Wu, Xiaoqiong Li, Xiangyu Bian, Jian Kuang, Jianqiang Li, Fangshu Shi, Ying Luo, Peiqing Jiang, Jinjun Li, Haibiao Sun

**Affiliations:** 1 The First Hospital of Shanxi Medical University, Taiyuan, China; 2 State Key Laboratory for Quality and Safety of Agro-Products, Institute of Food Sciences, Zhejiang Academy of Agricultural Sciences, Hangzhou, China; 3 Global R&D Innovation Center, Inner Mongolia Mengniu Dairy (Group) Co. Ltd., Hohhot, China

**Keywords:** *B. lactis* Ca360, calcium, *in vitro* fermentation, iron, mineral absorption, zinc

## Abstract

**Introduction:**

Mineral deficiency is a major nutritional issue that threatens human health. Probiotics, owing to their ability to enhance intestinal absorption, are regarded as potential nutritional modulators.

**Methods:**

In this study, multiple strains of *Lactobacillus* and *Bifidobacterium* were systematically evaluated for their *in vitro* fermentation metabolism and mineral absorption-promoting properties to screen probiotic candidates possessing mineral uptake-enhancing potential. Eight strains selected via multi-parameter screening were further evaluated for their mineral absorption-promoting capacity using the Caco-2 cell model.

**Results:**

The results revealed significant strain-specific differences in acid production capacity, short-chain fatty acids (SCFAs) generation, and phytase activity. Strains *L. paracasei* PC-01, *B. lactis* Ca360, *L. plantarum* Fe-01, *B. lactis* MN16620, and *L. brevis* MN14440 exhibited pronounced acid-producing ability, indicated by markedly decreased fermentation broth pH values. *L. reuteri* MN11965, *L. acidophilus* MN06785, *L. brevis* MN06618, and *L. rhamnosus* MN08244 showed significantly higher L-lactic acid yields than the positive control. Acetate was the predominant metabolite, followed by propionic and butyric acids, with *L. curvatus* MN15933, *B. lactis* Ca360, and *B. lactis* MN16620 showing particularly strong butyrate production. Phytase activity assays revealed that both intracellular and extracellular enzyme activities of *L. plantarum* Fe-01 and *B. lactis* Ca360 were significantly higher than those of *L. plantarum* 299v. In the Caco-2 cell model, all tested strains significantly increased calcium uptake, with *L. plantarum* Fe-01 and *B. lactis* Ca360 showing the highest transmembrane calcium transport efficiency. These two strains also markedly enhanced iron absorption, while *B. lactis* Ca360 exhibited zinc uptake and transport levels comparable to the positive control.

**Discussion:**

Comprehensive analysis indicated that strain *B. lactis* Ca360 demonstrated the most prominent effect in promoting calcium, iron, and zinc absorption, likely through mechanisms involving acid production-induced pH reduction and phytate hydrolysis facilitation. This study provides systematic verification of the integrated mechanisms by which probiotics promote mineral absorption and offers both theoretical support and strain resources for the development of targeted probiotics aimed at improving mineral bioavailability.

## 1 Introduction

Calcium (Ca), iron (Fe), and zinc (Zn) are indispensable for the growth, development, and metabolism of animals and humans. Micronutrient deficiency can lead to metabolic disorders and immune dysfunction in the body (Cannas et al., 2020; Dubey et al., 2020). Due to insufficient intake of dairy products, increased consumption of coffee and carbonated beverages, and excessive protein intake in some regions, more than half of the world's population suffers from insufficient dietary calcium intake (Shlisky et al., 2022). Calcium deficiency may cause diseases such as osteoporosis, gestational hypertension, and colorectal adenoma (Cormick and Belizán, 2019). Iron deficiency is also widespread, affecting more than 60% of the global population, and is therefore considered one of the most common micronutrient deficiencies worldwide (Cappellini et al., 2022; Leung et al., 2024). In addition to causing anemia, iron deficiency can lead to various clinical problems and functional impairments (Pasricha et al., 2021; Benson et al., 2022). Zinc deficiency is also one of the most prevalent micronutrient deficiencies globally, which can result in growth disorders, systemic immune decline, and neurobehavioral abnormalities, among other symptoms or signs with no obvious characteristics (Gupta et al., 2020; Wessels et al., 2021).

Current primary intervention strategies for mineral deficiencies encompass mineral supplements, dietary fortification, and pharmacological treatments. Mineral supplements such as calcium carbonate, ferrous sulfate, and zinc gluconate can directly elevate mineral levels within the body. However, their bioavailability is generally low, and excessive intake may lead to adverse effects, including gastrointestinal discomfort, constipation, nausea, kidney stones, and oxidative stress (Anselmo and Driscoll, 2021; Myung et al., 2021; Reid, 2025). Dietary fortification can partially improve population nutritional status, yet fortifying agents exhibit poor stability in the gastrointestinal environment. Furthermore, interactions with dietary components such as phytate, oxalate, and dietary fiber frequently diminish absorption efficiency (Cormick et al., 2021). Pharmaceutical interventions enhance absorption rates but entail high costs and may disrupt gut microbiota homeostasis with prolonged use, thereby affecting nutrient absorption and metabolic equilibrium (Finlayson-Trick et al., 2023; Karamantziani et al., 2024). Consequently, traditional supplementation approaches suffer from low absorption efficiency, poor physiological tolerance, and safety concerns, necessitating the development of a novel, safe, efficient, and sustainable strategy to enhance mineral absorption.

In recent years, probiotics and other microecological preparations have demonstrated significant potential in nutritional intervention and metabolic regulation. Research indicates that probiotics can promote mineral absorption through multiple mechanisms: acid-producing strains (such as lactobacilli and bifidobacteria) secrete lactic acid and acetate, lowering intestinal pH to enhance the solubility of ions including calcium, iron, and zinc (Varvara and Vodnar, 2024; Chatterjee et al., 2025); certain strains possess phytase activity, degrading phytate complexes to release chelated minerals (Bermudez-Humaran et al., 2024); probiotic fermentation of dietary fiber produces short-chain fatty acids (SCFAs), which not only further lower pH but also improve intestinal epithelial structure and tight junction protein expression, thereby enhancing trans-epithelial transport efficiency (Samtiya et al., 2021). Compared to traditional mineral supplements, microecological preparations offer the advantages of high safety and sustained regulatory effects, making them an ideal strategy for promoting mineral absorption. Among them, the genera *Lactobacillus* and *Bifidobacterium* are the most representative probiotics, and they have shown significant potential in the prevention and treatment of diseases such as inflammatory bowel disease, obesity, depression, and osteoporosis (Fikri et al., 2022). The mineral absorption-promoting effect of probiotics has been fully confirmed in *in vitro* and *in vivo* (animal or clinical trials) studies (Zhou et al., 2023; Harahap et al., 2024; Hu et al., 2024; Koker et al., 2024). Accordingly, we selectively selected *L. paracasei, L. plantarum, L. fermentum, L. reuteri, L. acidophilus*, and *B*. *Lactis, L. sakei, L. helveticus, L. rhamnosus*, and *L. curvatus* from the strain bank for subsequent screening.

This study aimed to identify probiotic strains capable of efficiently promoting mineral absorption (calcium, iron, zinc) through a multidimensional screening approach combining *in vitro* fermentation systems with the Caco-2 cell model. An integrated evaluation system linking acidification, enzyme activity, and transport facilitation was established by comprehensively assessing strains' acid production capacity, phytase activity, and short-chain fatty acid generation capability. This research provides experimental evidence elucidating the biological mechanisms by which probiotics enhance mineral absorption. It also offers a theoretical foundation and technical support for developing functional probiotic formulations targeting mineral absorption, holding significant scientific and practical value for addressing mineral absorption deficiencies globally.

## 2 Materials and methods

### 2.1 Source of experimental strains

The test strains were provided by China Mengniu Dairy (Group) Co., Ltd, Hohhot, Inner Mongolia, China. These probiotics had different acid-producing potentials as reported in existing literature, and were selected from diverse sources (children's intestines, infants' intestines, intestines of long-lived elderly, feces of healthy adults, traditional fermented products, naturally fermented cow's milk, naturally fermented soybean milk, yak milk, pickled cucumbers, spicy cabbage, sausages, etc.) with probiotic potential. Among them, 92% were lactic acid bacteria, including *Lactobacillus paracasei* (*L. paracasei*), *Lactobacillus plantarum* (*L. plantarum*), *Lactobacillus fermentum* (*L. fermentum*), *Lactobacillus reuteri* (*L. reuteri*), *Lactobacillus acidophilus* (*L. acidophilus*), *Lactobacillus sakei* (*L. sakei*), *Lactobacillus helveticus* (*L. helveticus*), *Lactobacillus rhamnosus* (*L. rhamnosus*), and *Lactobacillus curvatus* (*L. curvatus*); the remaining were four strains of *Bifidobacterium animalis* subsp. *Lactis* (*B. lactis*).

### 2.2 *In vitro* fermentation experiments

#### 2.2.1 Bacterial culture

The above strains were cultured in 5 mL of modified MRS medium (Haibo Biology, China) at 37 °C for 24 h; anaerobic bacteria were incubated under anaerobic conditions at 37 °C for 24 h for resuscitation and subculture. The strains were activated for 3 or more generations before use in experiments.

#### 2.2.2 *In vitro* fermentation

The *in vitro* batch fermentation system was modified with reference to the method of Chen et al. (2019). To simulate the starch-based dietary structure of Chinese residents, soluble starch (STA, Sigma-Aldrich) was added to the modified MRS medium at a final concentration of 8 g/L as the sole carbon source. The medium was adjusted to pH 6.5, dispensed into 10 mL vials (5 mL per vial), and sterilized by autoclaving at 121 °C for 15 min for later use. 150 μL of the test bacterial suspension (10^7^−10^8^ CFU/mL) was inoculated; *L. plantarum* 299v, possessing mineral absorption-promoting properties, was employed as the positive control. Add an equal volume of sterile medium to the blank control group. All samples were incubated in a constant-temperature anaerobic incubator at 37 °C for 24 h. After fermentation, the pH was measured, and the samples were aliquoted and stored at −80 °C for subsequent detection.

#### 2.2.3 Detection of L-lactic acid (L-LA) concentration

The content of L-lactic acid in the fermentation broth of each test strain was detected using a commercial kit (Beijing Solarbio Science & Technology Co., Ltd, China) according to the instructions.

#### 2.2.4 Detection of SCFAs concentration

Fermentation broth was centrifuged at 6,000 rpm for 10 min at 4 °C using a Heraeus Multifuge X1R refrigerated centrifuge (Thermo Fisher Scientific, Waltham, MA, USA). Two hundred fifty microliters of the supernatant was mixed with 50 μL crotonic acid solution (10 mM; Sigma-Aldrich, St. Louis, MO, USA), filtered through a 0.22 μm PTFE membrane (Millipore, Burlington, MA, USA), and stored at −80 °C for 24 h. SCFAs concentrations were determined using a GC-2010 Plus gas chromatograph equipped with a flame-ionization detector (Shimadzu, Japan) and a DB-FFAP column (30 m × 0.25 mm × 0.50 μm; Agilent, Santa Clara, CA, USA). Helium (≥99.999%) served as the carrier gas at 1.2 mL min^−1^, with an injector temperature of 300 °C, split ratio 10:1, and an oven program of 100 °C (0 min) → 250 °C at 20 °C min^−1^, giving a total run time of approximately 8 min. The FID was set at 300 °C with H_2_ 30 mL min^−1^, air 400 mL min^−1^, and He make-up 25 mL min^−1^. Calibration curves (0.05–10 mM) were prepared using mixed standards of acetate, propionate, isobutyrate, butyrate, isovalerate, and valerate (Sigma-Aldrich, USA) with crotonic acid as the internal standard. Quantification was based on analyte-to-internal-standard peak-area ratios, and data were processed with GCsolution software (Shimadzu, Japan). Method reproducibility was confirmed with calibration coefficients *R*^2^ ≥ 0.995 and RSD <5 %, and the FFAP column was conditioned daily at 240–250 °C for 20 min to maintain stability.

#### 2.2.5 Detection of phytase

Take 100 μL of the bacterial suspension to be tested (10^7^−10^8^ CFU/mL) and inoculate it into 10 mL of modified MRS medium. Incubate by shaking at 37 °C and 200 rpm for 24 h. Collect the culture medium, subject it to three cycles of liquid nitrogen freezing and room temperature thawing, then centrifuge at 4 °C and 8,000 rpm for 10 min. Collect the supernatant and bacterial pellet. Determine phytase activity using the molybdenum blue colourimetric method, following the instructions provided in the phytase detection kit (Beijing Bioscience Technology Co., Ltd., Beijing, China).

### 2.3 Cell experiments

#### 2.3.1 Source and culture of Caco-2 cells

Caco-2 human colon adenocarcinoma cells (Procell, China) were cultured in Dulbecco's Modified Eagle Medium (DMEM, high glucose, Sevier, China). The basic medium was supplemented with 10% (v/v) fetal bovine serum (FBS, Gibco, USA) and 1% (v/v) penicillin-streptomycin double antibody solution (Biyuntian, China). All experiments used cells from passages 10 to 25 (subcultured every 3 days), which were cultured in a constant-temperature incubator (Lide, Germany) at 37 °C with 5% CO_2_. When the cell confluency reached 80%−90%, the cells were digested and subcultured with 0.25% trypsin-EDTA (Biyuntian, China). The medium was replaced every 48 h.

#### 2.3.2 Detection of transepithelial transport capacity of calcium, iron, and zinc ions in Caco-2 monolayer cells

Caco-2 cells were seeded into the apical compartment of 12-well Transwell permeable supports (polyester membrane, pore size 0.4 μm, Jet Biofiltration, China) at a density of 5 × 10^5^ cells/well, and 1 mL of complete medium was added to the basolateral compartment. The cells were cultured for 15–20 days for differentiation in an incubator at 37 °C with 5% CO_2_ and 95% humidity. The transepithelial electrical resistance (TEER) of the Caco-2 monolayer was measured regularly using a Millicell resistance system (World Precision Instruments, Sarasota, FL, USA). The TEER value was calculated according to the following formula: TEER (Ω.cm^2^) = (R_t_-R_0_) × S, and a value greater than 400 Ω.cm^2^ was considered to indicate the formation of a complete monolayer. The calcium ion transport efficiency was determined with modifications based on the method of Raveschot et al. (2020). To prepare the bacterial suspension for the transport assay, bacterial strains were cultured as described in Section 2.2.1. The cells were harvested by centrifugation at 6,000 rpm for 10 min at 4 °C and washed twice with sterile phosphate-buffered saline (PBS, pH 7.4) to remove residual MRS medium. The bacterial pellets were then resuspended in cell culture medium (DMEM without FBS and antibiotics) and adjusted to an optical density (OD600) of 1.0, corresponding to a concentration of approximately 1 × 10^8^ CFU/mL (Fonseca et al., 2021). This standardized bacterial suspension was used immediately for the co-culture experiments. Separate experiments were conducted to determine calcium, iron, and zinc absorption. The Caco-2 monolayers were washed twice with DPBS, and 500 μL of bacterial suspension (or DMEM control) was mixed with 10 μL of the specific mineral substrate—CaCl_2_ (250 mM) (Raveschot et al., 2020), iron (FeSO4, 50 μM), or zinc (ZnSO4, 50 μM) (Shilpashree et al., 2020), respectively, and added to the apical compartment of the cells. After incubation at 37 °C with 5% CO_2_ for 24 h, the medium in the basolateral compartment was collected, filtered through a 0.22 μm filter membrane, and the transport amounts of calcium, iron, and zinc were measured using an inductively coupled plasma optical emission spectrometer (Jena, Germany). The transport efficiency was calculated as the percentage of the mineral amount in the basolateral chamber relative to the total initial amount added to the apical chamber, using the formula: Transport Efficiency (%) = (Amount in basolateral chamber / Total initial amount) × 100%.

#### 2.3.3 Detection of uptake capacity of calcium, iron, and zinc ions in Caco-2 monolayer cells

With modifications based on the method of Krupa-Kozak et al. (2016), after completing the calcium, iron, and zinc ion transport experiments, the cell monolayer was gently washed with pre-cooled HBSS (4 °C, pH 7.4), subjected to 3 cycles of liquid nitrogen freezing and room temperature thawing, and then centrifuged at 12,000 rpm at 4 °C for 15 min. The supernatant was collected, and the intracellular contents of calcium, iron, and zinc were also measured using an inductively coupled plasma optical emission spectrometer. The cellular uptake efficiency was calculated as the percentage of the mineral amount retained within the cell monolayer relative to the total initial amount added to the apical chamber, using the formula: Cellular Uptake Efficiency (%) = (Amount in cell lysate / Total initial amount) × 100%.

### 2.4 Statistical analysis of data

All data in this study were analyzed using GraphPad Prism 10.1.2. Unless otherwise stated, all experiments were performed in triplicate as independent biological replicates (*n* = 3). Data distribution was assessed for normality using the Shapiro-Wilk test, and homogeneity of variance was verified using Brown test. Differences between groups were analyzed using One-way Analysis of Variance (ANOVA) followed by Tukey's multiple comparison test, the non-parametric. Kruskal-Wallis test followed by Dunn's multiple comparison test was applied. Since fermentation characteristics and mineral transport assays represent distinct physiological processes, these endpoints were analyzed independently, with Tukey's test applied to control the family-wise error rate within each dataset. Data are expressed as mean ± standard error of the mean (SEM), and a *P*-value <0.05 was considered statistically significant.

## 3 Results

### 3.1 Results of *in vitro* fermentation

#### 3.1.1 pH changes of different strains after *in vitro* fermentation

**Figure 1:**
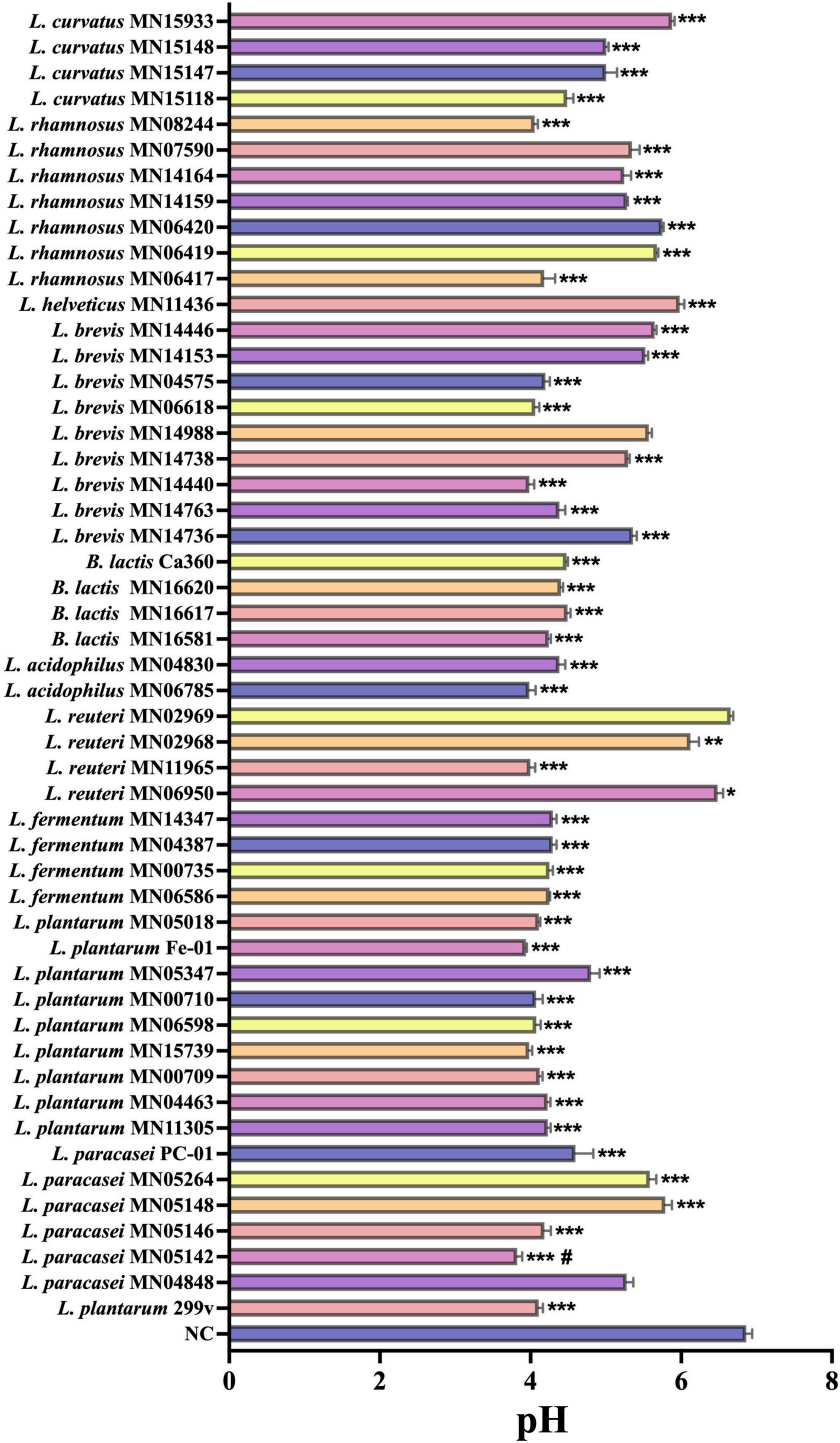
Changes in pH of fermentation broths of different strains after *in vitro* fermentation, *n* = 3. *P < 0.05; **P < 0.01; ***P < 0.001 vs. NC; ^#^*P* < 0.05 vs. *L. plantarum* 299v.

Figure 1 displays the pH test results. The *in vitro* fermentation results showed that the fermentation broth pH values of strains *L. paracasei* PC-01, *B. lactis* Ca360, *L. plantarum* Fe-01, *B. lactis* MN16620, *L. brevis* MN14440, and *L. plantarum* Fe-01 were significantly lower than those of the control group (NC). This finding indicates that these probiotic strains possess strong acid-producing capacities during *in vitro* fermentation, effectively decreasing the pH of the culture system. Such acidification contributes to the establishment of an intestinal-like acidic environment, thereby creating favorable conditions for the subsequent exertion of probiotic functions.

#### 3.1.2 L-LA producing ability of test strains

**Figure 2:**
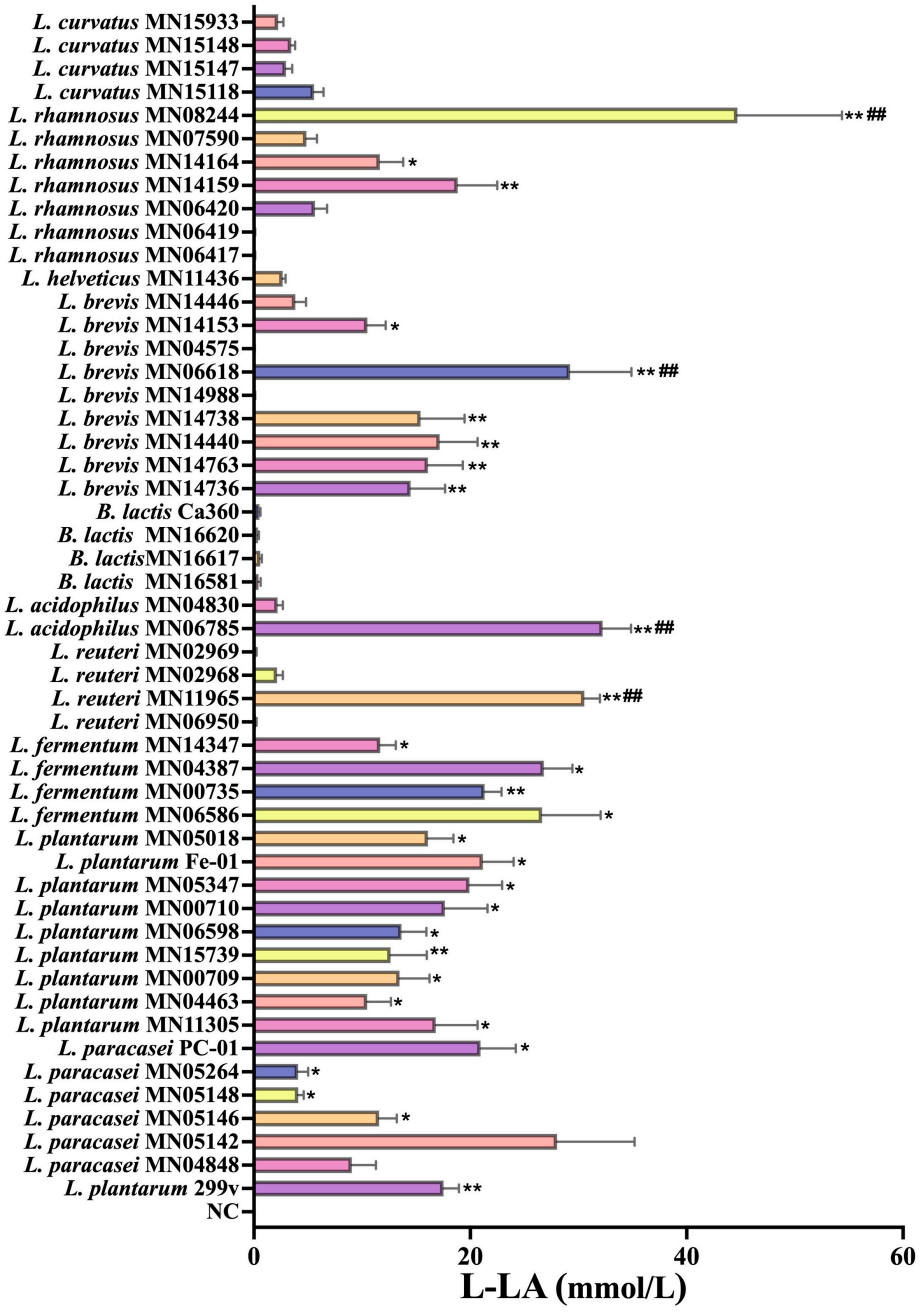
L-LA producing ability of test strains, *n* = 3. *P < 0.05; **P < 0.01 vs. NC; ^##^*P* < 0.05 vs. *L. plantarum* 299v.

The determination of L-lactic acid (L-LA) production is shown in Figure 2. The L-LA yields of *L. reuteri* MN11965, *L. acidophilus* MN06785, *L. brevis* MN06618, and *L. rhamnosus* MN08244 exhibited significantly higher L-LA yields than the positive control strain *L. plantarum* 299v, indicating their stronger lactic acid synthesis and metabolic capacities. Meanwhile, the L-LA yields of strains *L. paracasei* PC-01, *L. plantarum* Fe-01, *L. fermentum* MN06586, and *L. fermentum* MN04387 were comparable to those of *L. plantarum* 299v (*P* > 0.05), suggesting that these strains maintain a stable acid-producing profile. Overall, the majority of the tested strains demonstrated robust lactic acid production during *in vitro* fermentation, supporting their potential as key candidates for subsequent functional evaluation.

#### 3.1.3 Detection and analysis of SCFAs-producing ability of test strains

**Figure 3:**
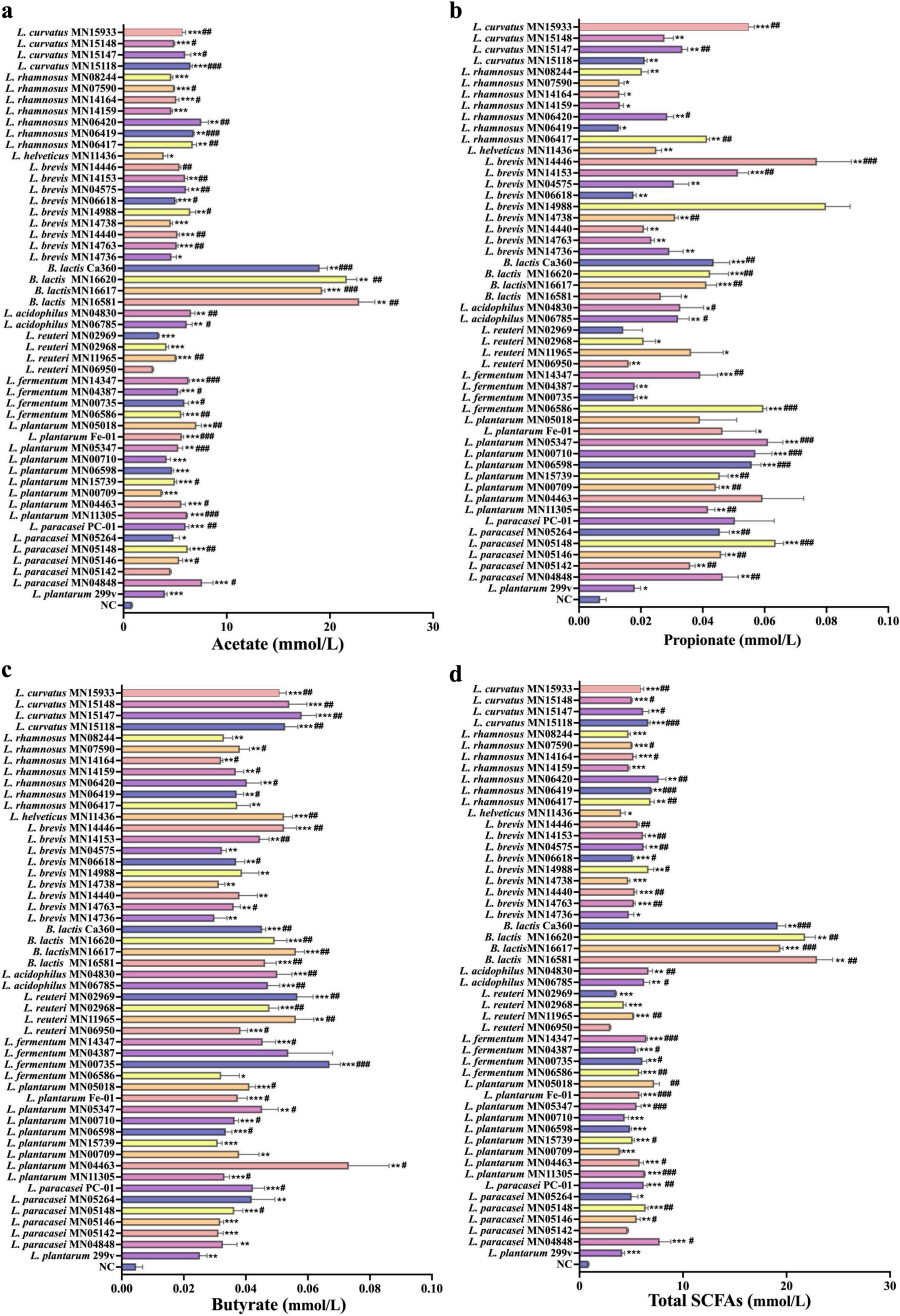
SCFAs-producing ability of test strains. **(a)** Acetate; **(b)** Propionate; **(c)** Butyrate; **(d)** Total SCFAs, *n* = 3. *P < 0.05; **P < 0.01; ***P < 0.001 vs. NC; ^#^*P* < 0.05; ^##^*P* < 0.01; ^###^*P* < 0.001 vs. *L. plantarum* 299v.

Short-chain fatty acids (SCFAs) are important metabolites of probiotics, known to play key roles in enhancing immunity and providing energy for intestinal epithelial cells. In this study, the SCFAs content in the fermentation broths of different Lactobacillus strains was quantitatively assessed. As shown in Figures 3a–d, significant strain-specific differences in SCFAs yields were observed. Overall, acetate was the predominant product, followed by propionate and butyrate, with trace amounts of isobutyrate, valerate, and isovalerate detected in some strains. In terms of acetate production, all tested *B.lactis* strains exhibited superior acid-producing capacities (Figure 3a). Additionally, strains such as *L. paracasei* PC-01, *L. paracasei* MN04848, *L. plantarum* MN05018, and *L. plantarum* Fe-01 demonstrated better acetate production than the positive control, *L. plantarum* 299v. For propionate, strains *L. curvatus* MN15933, *L. brevis* MN14446, *L. brevis* MN14153, *B. lactis* Ca360, and *B. lactis* MN16620 showed significantly higher yields than *L. plantarum* 299v, suggesting these strains possess strong carbon source conversion capabilities and enhanced propionate production (Figure 3b). Butyrate assays revealed that strains *L. curvatus* MN15147, *L. curvatus* MN15933, *B. lactis* Ca360, *B. lactis* MN16620, *L. plantarum* Fe-01, and *L. paracasei* PC-01 exhibited significantly higher butyrate yields than *L. plantarum* 299v, indicating these strains have a strong butyrate conversion potential (Figure 3c). The production of valerate and branched-chain fatty acids was generally low, showing no significant differences compared to *L. plantarum* 299v, as detailed in Supplementary Figure S1.

#### 3.1.4 Screening results of the phytase-producing ability of test strains

**Figure 4:**
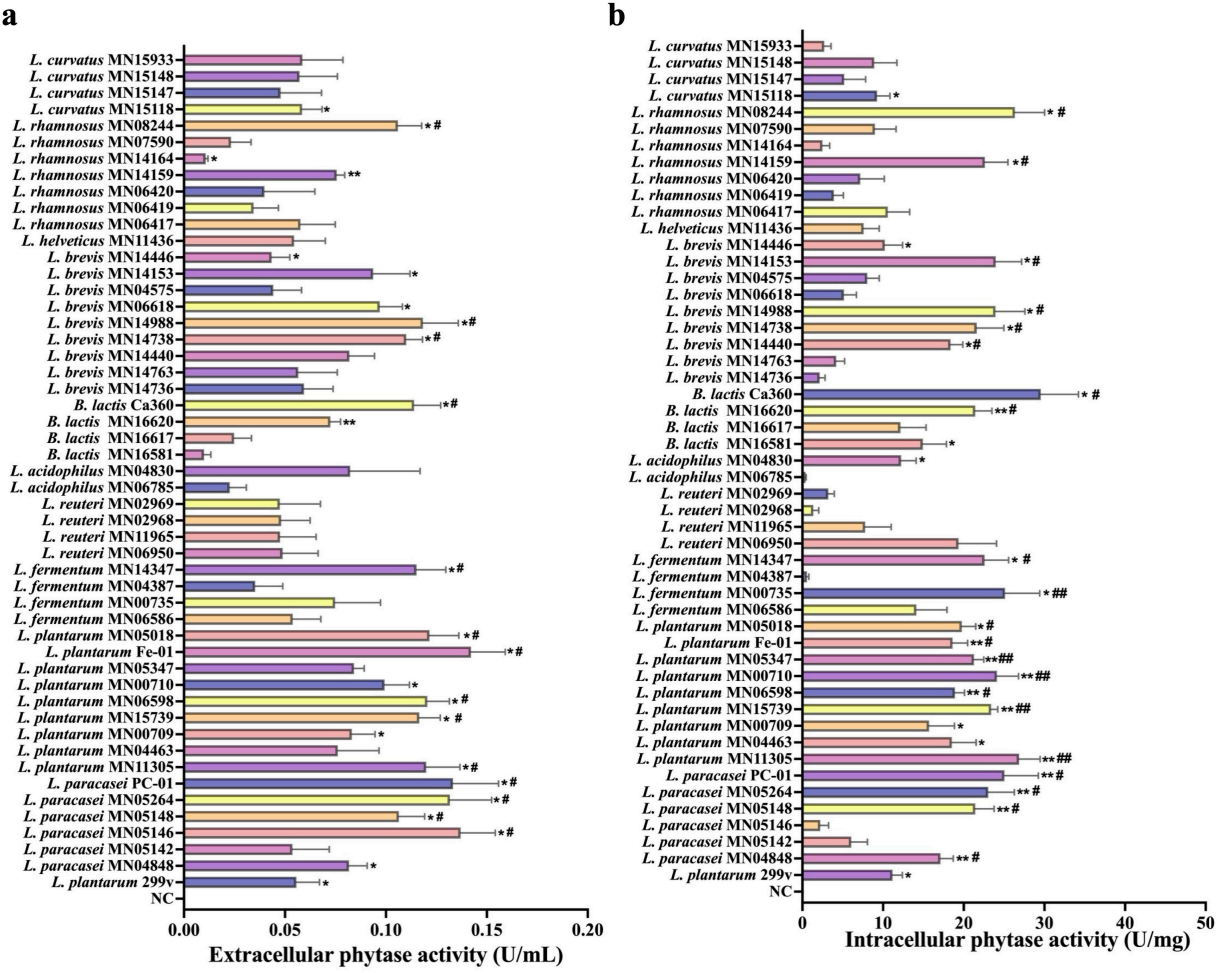
Phytase-producing ability of test strains. **(a)** Extracellular phytase activity. **(b)** Intracellular phytase activity. *n* = 3. *P < 0.05; **P < 0.01 vs. NC; ^#^*P* < 0.05; ^##^*P* < 0.01 vs. *L.plantarum* 299v.

Phytase plays a crucial role in promoting mineral absorption by probiotics, as it degrades phytate and releases minerals (such as calcium, iron, zinc, etc.) from plant-based foods, enhancing their bioavailability and thereby facilitating intestinal mineral absorption. To further investigate the mineral absorption–promoting capacity of probiotics, we evaluated their intracellular and extracellular phytase production abilities. As shown in Figure 4a, significant strain-specific differences in extracellular phytase activity were observed among the probiotics. Overall, most strains did not exhibit significant differences in extracellular phytase activity compared to the positive control strain, *L. plantarum* 299v. However, several strains, including *L. paracasei* PC-01, *L. paracasei* MN05264, *L. paracasei* MN05146, *L. plantarum* MN05018, *L. plantarum* Fe-01, and *L. plantarum* MN06598, showed significantly higher extracellular phytase activities than *L. plantarum* 299v, indicating a stronger phytate-degrading ability. Among these, *L. plantarum* Fe-01 exhibited the highest extracellular phytase activity. Intracellular phytase activity assays, shown in Figure 4b, revealed that strains including *L. rhamnosus* MN08244, *L. rhamnosus* MN14159, *B. lactis* Ca360, *B. lactis* MN16620, *L. plantarum* MN05018, *L. plantarum* Fe-01, and *L. plantarum* MN06598 displayed significantly higher intracellular phytase activities compared to *L. plantarum* 299v, demonstrating enhanced phytase production capabilities.

### 3.2 Effects of different test strains on calcium absorption and transport capacity of Caco-2 cells

**Figure 5:**
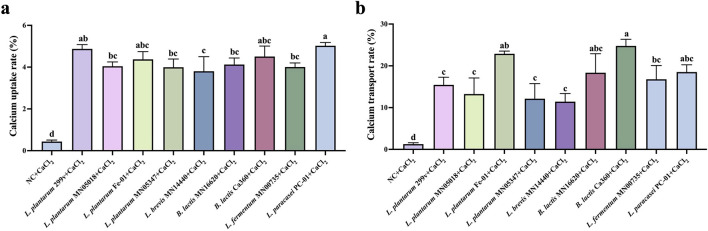
Effects of different test strains on calcium uptake and transport capacity of Caco-2 cells. **(a)** Calcium uptake rate; **(b)** Calcium transport rate, *n* = 3. There exists a statistically significant difference between different letters, *P* < 0.05.

Based on the previous multi-parameter screening (acid production/SCFAs, phytase activity, etc.), strains *L. plantarum* MN05018, *L. plantarum* Fe-01, *L. fermentum* MN00735, *L. brevis* MN14440, *B. lactis* Ca360, *L. plantarum* MN05347, and *L. paracasei* PC-01 were selected for further validation. The effect of these strains chosen on calcium uptake and transmembrane transport was evaluated using the Caco-2 monolayer model. The experimental results are shown in Figure 5. All selected strains significantly increased intracellular calcium uptake compared to the negative control group (NC), with no statistical differences observed when compared to *L. plantarum* 299v, indicating that these strains overall possess calcium uptake capabilities comparable to those of classical probiotics (Figure 5a). In the study of calcium transmembrane transport, *L. plantarum* Fe-01 and *B. lactis* Ca360 exhibited significantly higher calcium flux from the apical to the basolateral side compared to *L. plantarum* 299v, while the other strains showed no significant differences (Figure 5b). These results suggest that *L. plantarum* Fe-01 and *B. lactis* Ca360 are particularly effective in promoting calcium transmembrane transport. In summary, *L. plantarum* Fe-01 and *B. lactis* Ca360 demonstrate excellent potential in enhancing calcium absorption in the intestine.

### 3.3 Effects of different test strains on iron absorption and transport capacity of Caco-2 cells

**Figure 6:**
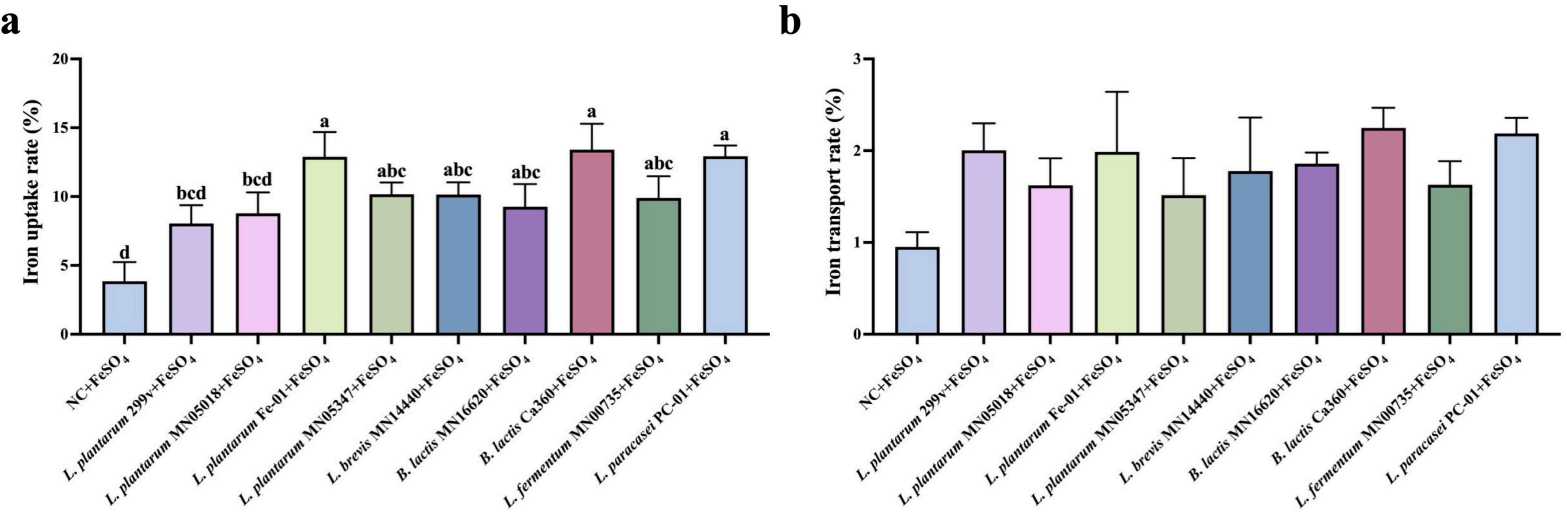
Effects of different test strains on iron uptake and transport capacity of Caco-2 cells. **(a)** Iron uptake rate; **(b)** Iron transport rate, *n* = 3. There exists a statistically significant difference between different letters, *P* < 0.05.

Figure 6 illustrates the effect of probiotics on iron uptake and transmembrane transport in the Caco-2 monolayer model. As shown in Figure 6a, *L. plantarum* Fe-01 and *B. lactis* Ca360 exhibited significantly higher iron uptake rates compared to the positive control, suggesting that these two strains have a stronger ability to promote iron absorption, while no significant differences were observed between the other strains and the positive control. Additionally, the transmembrane transport experiment results indicated that the intracellular iron transport rates of all selected strains were comparable to that of the positive control, *L. plantarum* 299v, with no statistical differences (Figure 6b). This suggests that the strains chosen exhibit transmembrane iron transport capabilities similar to *L. plantarum* 299v. Overall, the results demonstrate that the selected strains generally enhance iron uptake, with *L. plantarum* Fe-01 and *B. lactis* Ca360 showing the most prominent effects during the iron absorption process, highlighting their potential application value.

### 3.4 Effects of different test strains on zinc absorption and transport capacity of Caco-2 cells

**Figure 7:**
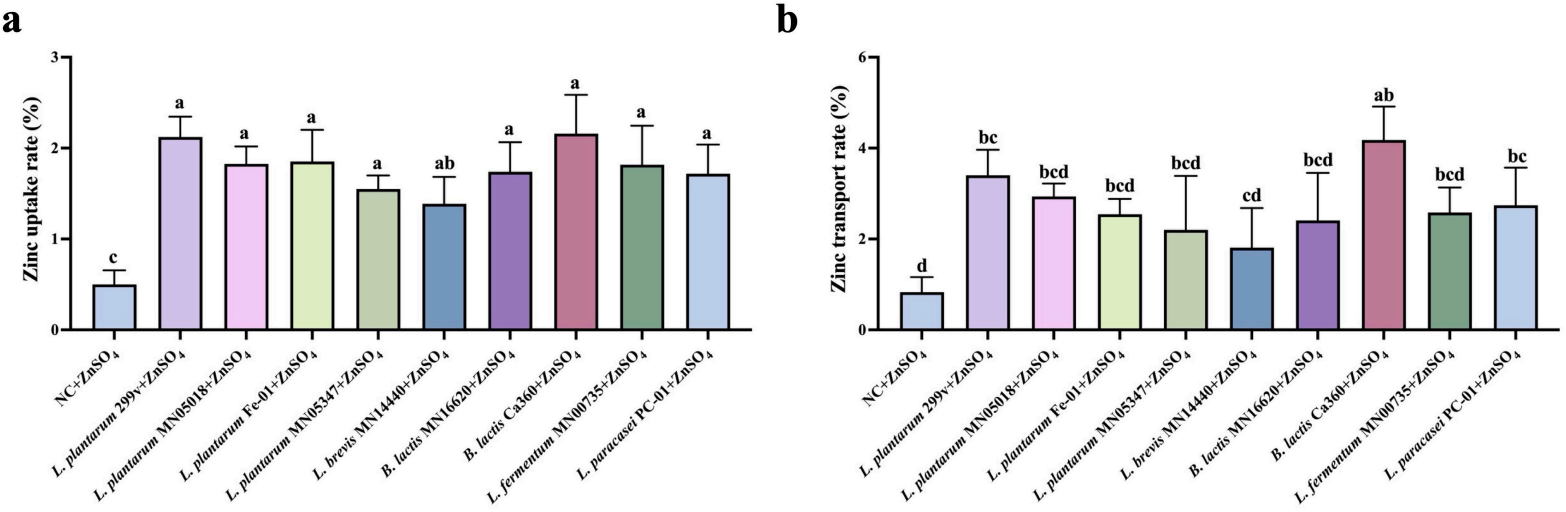
Effects of different test strains on zinc uptake and transport capacity of Caco-2 cells. **(a)** Zinc uptake rate; **(b)** Zinc transport rate, *n* = 3. There exists a statistically significant difference between different letters, *P* < 0.05.

Figure 7 illustrates the effects of probiotics on zinc uptake and transmembrane transport in the Caco-2 monolayer model. Overall, all probiotic treatment groups exhibited higher intracellular zinc uptake and transmembrane transport rates compared with the negative control group. However, no significant differences were observed when compared with the positive control strain, *L. plantarum* 299v, indicating that the selected strains generally possess a certain potential to enhance zinc absorption (Figure 7a). Among them, *B. lactis* Ca360 displayed zinc uptake and transmembrane transport rates comparable to those of the positive control (Figure 7b), suggesting that this strain exhibits a stable and favorable effect in promoting zinc uptake and transport across intestinal epithelial cells.

## 4 Discussion

This study systematically evaluated the metabolic and functional characteristics of multiple *Lactobacillus* and *Bifidobacterium* strains, with particular emphasis on their acid-producing capacity, phytase activity, and ability to promote mineral absorption under *in vitro* conditions. The results demonstrated pronounced strain-specific differences in fermentation profiles, which is consistent with the metabolic diversity widely reported among probiotic species (Bermudez-Humaran et al., 2024). The strong acidification capacity observed in strains such as *L. paracasei* PC-01, *L. plantarum* Fe-01, and *B. lactis* Ca360 indicates efficient carbohydrate metabolism, leading to the accumulation of lactic acid and SCFAs. These metabolites are known to modulate the intestinal microenvironment by lowering luminal pH, which may inhibit pathogenic colonization and enhancing nutrient solubility. In particular, SCFAs (acetate, propionate, and butyrate) are reported to play essential roles in epithelial energy metabolism and intestinal integrity through activation of GPR41/43 signaling and histone deacetylase inhibition (Liu et al., 2023; Du et al., 2024; Mann et al., 2024; Mukhopadhya and Louis, 2025). The variation in metabolite profiles among strains implies distinct substrate utilization and metabolic network adaptations, which may contribute to differential impacts on mineral bioavailability.

Among the evaluated strains, *B. lactis* Ca360 exhibited notably high intracellular and extracellular phytase activities, exceeding that of the benchmark probiotic *L. plantarum* 299v. Phytase catalyzes the hydrolysis of phytic acid, releasing bound minerals such as Ca^2+^, Fe^2+^, and ^2+^, thereby improving their solubility and intestinal uptake (Joudaki et al., 2023; Chondrou et al., 2024). Enhanced phytase production by *B. lactis* Ca360 and *L. plantarum* Fe-01 suggests a strong capacity to degrade phytate under simulated intestinal conditions, which may increase the availability of free mineral ions.

Functional evaluation using the Caco-2 cell monolayer model further supported the mineral-promoting potential of these strains under *in vitro* conditions (Jovaní et al., 2001; Perales et al., 2005). It should be noted that all functional observations in this study were obtained using *in vitro* models, and therefore do not fully represent the complexity of mineral absorption under physiological *in vivo* conditions. All selected strains enhanced calcium uptake relative to the negative control, while *L. plantarum* Fe-01 and *B. lactis* Ca360 demonstrated the most significant increases in transmembrane calcium transport. The observed enhancement may arise from multiple synergistic mechanisms, including environmental acidification facilitating calcium solubilization, chelation of Ca^2+^ by organic acid anions (lactate, citrate), and potential modulation of calcium transport proteins such as TRPV6, CaBP-D9k, and PMCA1b (Wang et al., 2022; Koker et al., 2024; Varvara and Vodnar, 2024). Additionally, SCFAs such as acetate and butyrate may indirectly promote calcium flux, potentially through effect on tight junction proteins and epithelial permeability. These findings collectively suggest that probiotic-derived metabolites may act as physiological modulators of calcium bioavailability.

In the case of iron absorption, *L. plantarum* Fe-01 and *B. lactis* Ca360 significantly increased intracellular iron uptake, consistent with prior evidence that lactic acid bacteria and bifidobacteria enhance non-heme iron absorption by acidifying the intestinal environment and reducing ferric to ferrous iron (Koker et al., 2024; Apte et al., 2025). Furthermore, bifidobacteria are known to express surface-associated reductases and exopolysaccharides capable of forming soluble Fe^2+^ complexes, which could further improve iron transport, potentially involving DMT1 and FPN pathways (Baruah et al., 2023; Wang et al., 2024). These complementary biochemical and transport mechanisms likely underpin the observed enhancement of iron uptake by *B. lactis* Ca360.

Although zinc absorption did not differ significantly among most strains, *B. lactis* Ca360 displayed zinc uptake and transmembrane transport efficiencies comparable to those of *L. plantarum* 299v. This result implies a stable ability to facilitate Zn^2+^ bioavailability, possibly via secretion of organic acids and exopolysaccharides that form soluble zinc complexes (Nishito et al., 2023; Varvara and Vodnar, 2024). Additionally, probiotic metabolites may contribute to zinc homeostasis at the epithelial interface, potentially involving zinc transporters such as ZnT1 and ZIP4. The stable and balanced performance of *B. lactis* Ca360 across calcium, iron, and zinc absorption pathways highlights its potential as a multifunctional mineral-regulating probiotic strain. Notably, *B. lactis* Ca360 demonstrated a distinct competitive advantage over the commercial benchmark *L. plantarum* 299v, particularly in extracellular phytase activity and calcium/iron transport efficiency. This suggests that strain selection based on this unique metabolic profile—specifically the synergy between robust acidification and high phytate hydrolysis—may offer superior therapeutic efficacy compared to existing commercial strains relying predominantly on general colonization.

However, these findings must be interpreted with caution. While the Caco-2 model and i*n vitro* fermentation systems effectively simulate the intestinal epithelial barrier, they represent simplified environments that lack the full physiological complexity of the gastrointestinal tract, including gastric digestion phases, mucosal immune interactions, and dynamic gut microbiota regulation. Consequently, the observed superior transport efficiency primarily validates our screening strategy rather than acting as a direct predictor of systemic efficacy. Future research requires validation in animal models and human studies to confirm these effects *in vivo*, as well as further investigation into the specific interactions among probiotic-derived metabolites not addressed in this study. Despite these limitations, the present work establishes a valuable screening framework for identifying probiotic strains with potential mineral absorption–promoting properties.

## 4 Conclusions

This study established an integrated screening system based on acid production capacity, SCFAs generation, and phytase activity to evaluate the potential of probiotics in promoting mineral absorption. The results demonstrated that several *Lactobacillus and Bifidobacterium* strains exhibited strong metabolic activity and phytate-degrading capacity during *in vitro* fermentation, contributing to improved mineral solubility and bioavailability. Validation using the Caco-2 cell model further revealed that *B. lactis* Ca360 exhibited the most pronounced enhancement of calcium, iron, and zinc absorption, indicating a multi-pathway promotion effect. Collectively, this study identified a functional probiotic strain—*B. lactis* Ca360—with significant potential to enhance mineral absorption, systematically elucidated key functional traits and screening strategies of mineral absorption–promoting probiotics, and provided both theoretical support and microbial resources for the development of functional probiotics with targeted mineral-regulatory capabilities.

## Data Availability

The original contributions presented in the study are included in the article/Supplementary material, further inquiries can be directed to the corresponding authors.
