# Correction: Screening of probiotics for promoting mineral absorption based on *in vitro* fermentation and cell models

**DOI:** 10.3389/fmicb.2026.1819293

**Published:** 2026-03-20

**Authors:** Bin Liu, Yuejian Mao, Jing Yang, Linjun Wu, Xiaoqiong Li, Xiangyu Bian, Jian Kuang, Jianqiang Li, Fangshu Shi, Ying Luo, Peiqing Jiang, Jinjun Li, Haibiao Sun

**Affiliations:** 1 The First Hospital of Shanxi Medical University, Taiyuan, China; 2 State Key Laboratory for Quality and Safety of Agro-Products, Institute of Food Sciences, Zhejiang Academy of Agricultural Sciences, Hangzhou, China; 3 Global R&D Innovation Center, Inner Mongolia Mengniu Dairy (Group) Co. Ltd., Hohhot, China

**Keywords:** *B. lactis* Ca360, calcium, *in vitro* fermentation, iron, mineral absorption, zinc

Author Bin Liu was erroneously spelled as Bing Liu.

The original version of this article has been updated.

