# Supplementary material for: Screening of probiotics for promoting mineral absorption based on in vitro fermentation and cell models
Source: Front Microbiol. 2026 Feb 26;17:1743657. doi: 10.3389/fmicb.2026.1743657 (PMC12979561; doi:10.3389/fmicb.2026.1743657)
Supplement: Supplementary file 1 [file Supplementary_file_1.docx]

Supplementary Material

## Supplementary Figures


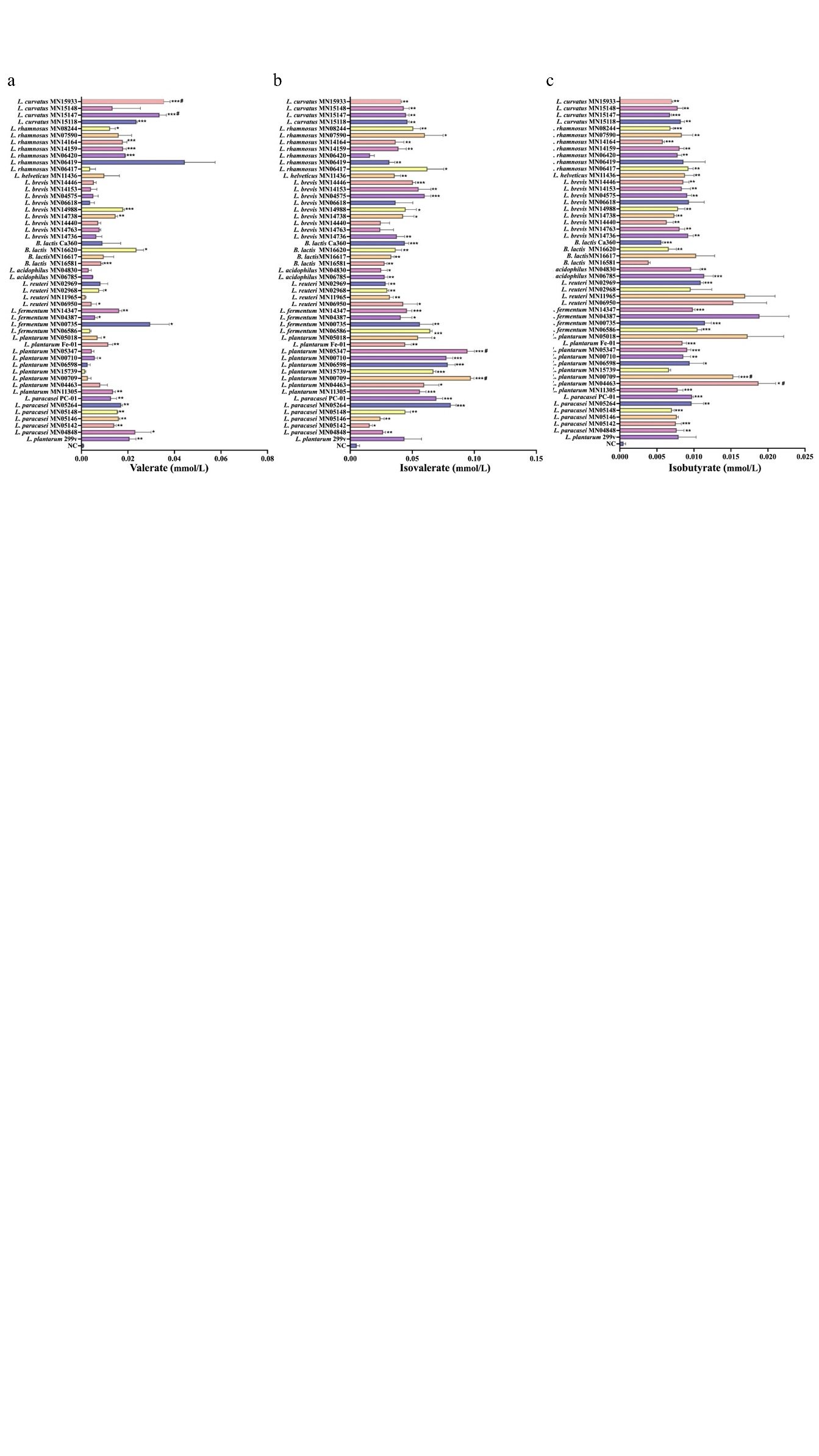


**Supplementary Figure 1.** SCFA-producing ability of test strains. (a) Valerate (b) Isovalerate; (c) Isobutyrate, n=3. **P* < 0.05; ***P* < 0.01; ****P* < 0.001 VS NC; ^#^*P* < 0.05; ^##^*P* < 0.01; ^###^*P* < 0.001 VS *L. plantarum* 299v.


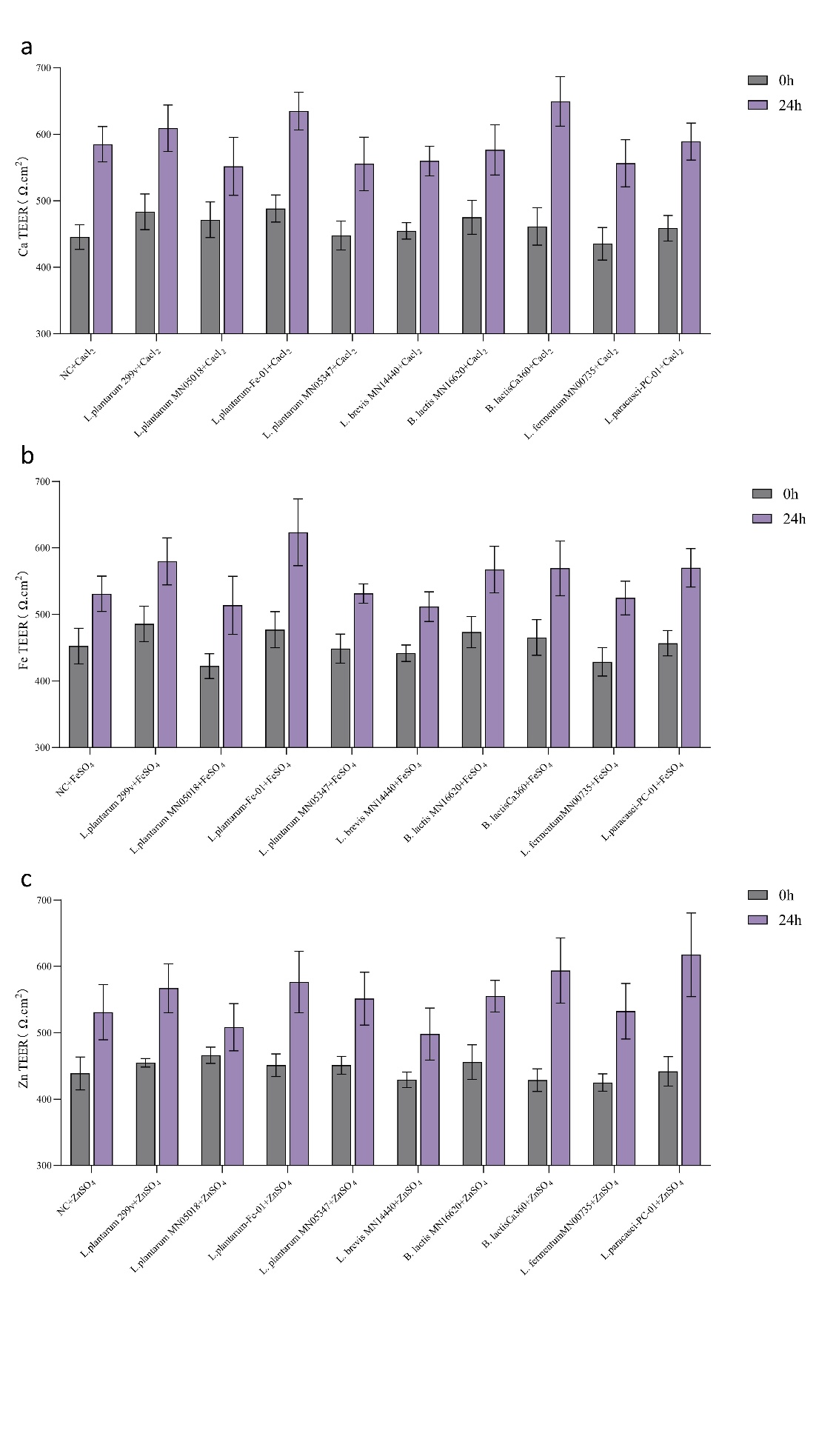


**Supplementary Figure 2.** Changes in *trans*-epithelial electrical resistance (TEER) across Caco-2 cell monolayers following incubation with probiotic strains and mineral supplements. TEER values (Ω⋅cm²) were measured at time t = 0 h (dark grey) and 24 h (purple). (a) TEER evolution in the presence of ZnSO_4_. (b) TEER evolution in the presence of FeSO_4_. (c) TEER evolution in the presence of CaCl_2_. Values are means ± SD of repeated measurements.
